# Worm Colic by Massive Biliary Ascariasis: A Case Report

**DOI:** 10.7759/cureus.113723

**Published:** 2026-07-31

**Authors:** Izzattaufiq Awang, Ikhwan Sani Mohamad, Mohd Azem Fathi Mohammad Azmi, Wan Muhamad Mokhzani Wan Muhamad Mokhter

**Affiliations:** 1 Surgery, Universiti Sains Malaysia School of Medical Sciences, Kota Bharu, MYS; 2 Surgery, Hospital Universiti Sains Malaysia, Kota Bharu, MYS

**Keywords:** ascaris lumbricoides, biliary ascariasis, biliary colic, endoscopic retrograde cholangiopancreatography (ercp), ultrasonography

## Abstract

Biliary ascariasis is a well-recognized hepatobiliary complication of infection with *Ascaris lumbricoides*. The disease remains highly prevalent in tropical and subtropical regions, particularly in Southeast Asia, where environmental and socioeconomic factors facilitate transmission. We report a 73-year-old female who presented with right hypochondriac pain with abdominal ultrasonography of biliary ascariasis, which was successfully managed with endoscopic retrograde cholangiopancreatography, extracting 14 *A. lumbricoides.*

## Introduction

Biliary ascariasis is a well-recognized hepatobiliary complication of infection with *Ascaris lumbricoides*, the largest intestinal nematode infecting humans [1]. The disease remains endemic in tropical and subtropical regions, particularly in Southeast Asia, where warm climatic conditions, poor sanitation, and low socioeconomic status contribute to continued transmission [1,2]. Individuals residing in rural communities and those engaged in agricultural occupations are at increased risk because of frequent exposure to contaminated soil [1-3].

Migration of adult worms from the small intestine into the biliary tree may result in biliary obstruction, presenting as biliary colic, obstructive jaundice, acute cholangitis, pancreatitis, or hepatic abscess formation [1]. Abdominal ultrasonography is the preferred first-line imaging modality owing to its accessibility, high diagnostic accuracy, and ability to demonstrate motile intraductal worms. When ultrasonography is inconclusive, magnetic resonance cholangiopancreatography (MRCP) or computed tomography (CT) may provide additional diagnostic information. Endoscopic retrograde cholangiopancreatography (ERCP) remains the gold standard for both diagnosis and treatment in patients with biliary obstruction, while antihelminthic therapy is recommended following biliary clearance to eradicate residual intestinal infestation and reduce recurrence [3-9].

Although biliary ascariasis is frequently encountered in endemic regions, recurrent disease following previous ERCP with an exceptionally heavy worm burden is rarely reported, particularly in elderly patients after cholecystectomy. We report a case of recurrent massive biliary ascariasis in a post-cholecystectomy elderly woman from a rural endemic setting, successfully managed with standard ERCP despite the extraction of 14 *A. lumbricoides*. This case highlights that even in the presence of extensive biliary infestation, established diagnostic and therapeutic principles remain effective.

## Case presentation

A 73-year-old female with a history of laparoscopic cholecystectomy presented with right upper quadrant pain radiating to the epigastric region for three days. She denied fever, vomiting, jaundice, or changes in bowel habits. There was no history of traditional medication use. She is involved in agricultural activities, including farming and livestock rearing.

She had two prior admissions for biliary ascariasis, during which she underwent ERCP, where sphincterotomy was performed, and successful extraction of *A. lumbricoides* from the common bile duct (CBD). She only completed a single dose of anti-helminthic during her previous admission.

**Figure 1:**
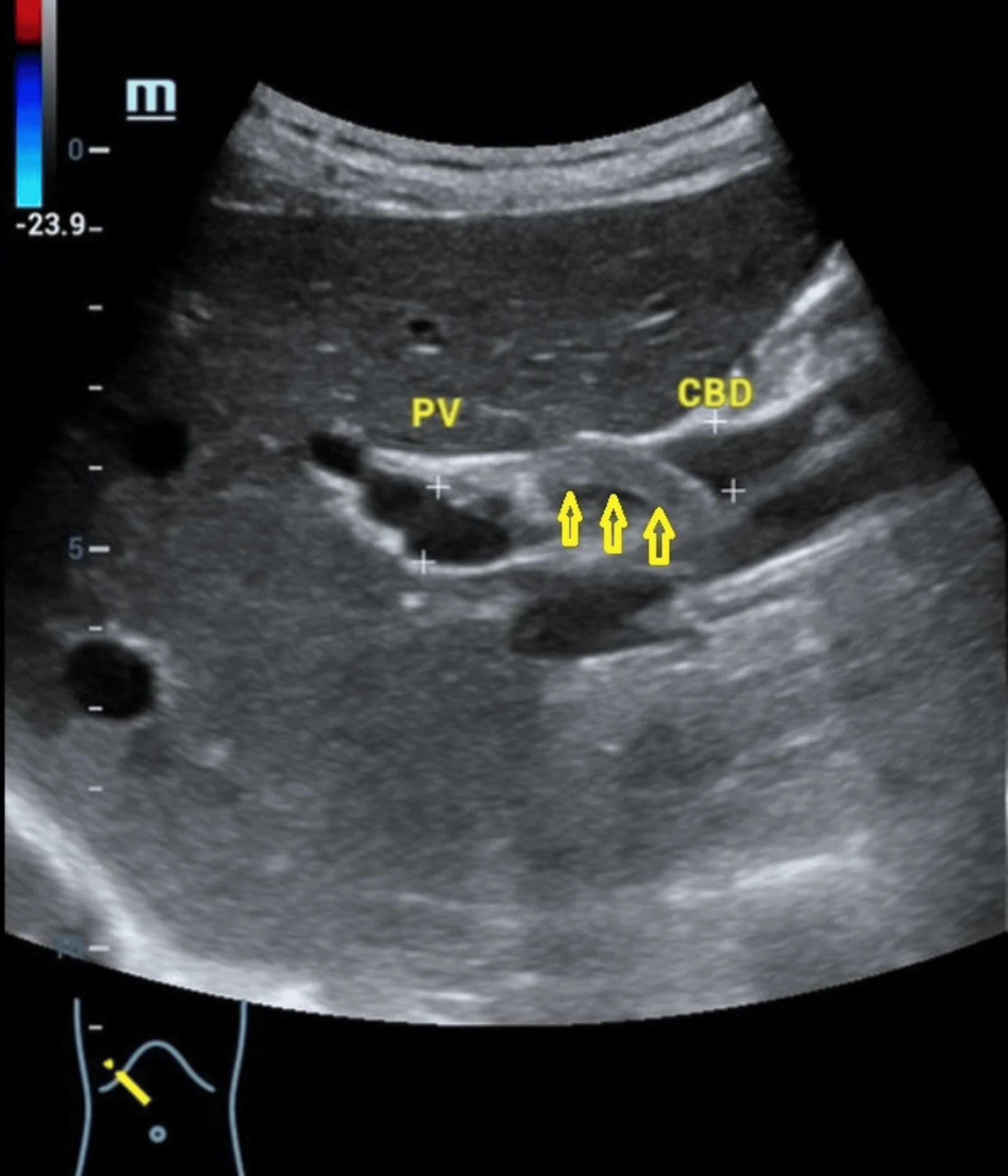
Ultrasound showing tubular hyperechoic structure in CBD The yellow arrows show *Ascaris lumbricodes.* CBD: common bile duct; PV: portal vein

On examination, she had muddy sclera, and her abdomen was soft and non-tender. Vital signs taken on arrival were within normal limits. Laboratory investigations showed mildly elevated liver enzymes with normal bilirubin levels (aspartate aminotransferase (AST): 113 U/L (10-35 U/L); alanine transaminase (ALT): 94 U/L (10-35 U/L); alkaline phosphatase (ALP): 189 U/L (35-104 U/L); total bilirubin: 4 µmol/L (<15)). Other blood investigations were normal. Ultrasonography revealed a tubular hyperechoic structure within the CBD, suggestive of biliary ascariasis (Figure 1).

**Figure 2:**
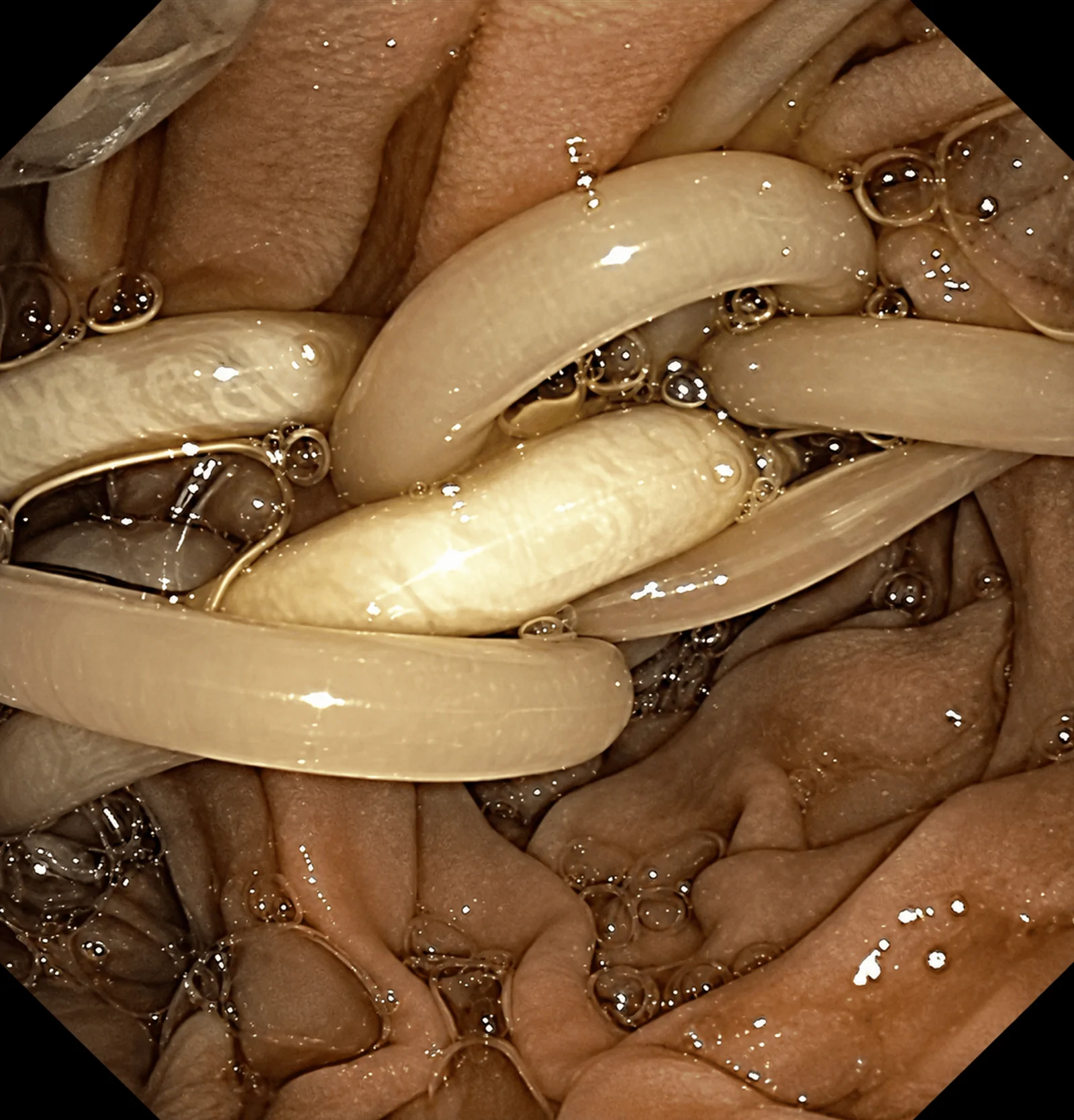
Clump of worms at the ampulla of Vater

**Figure 3:**
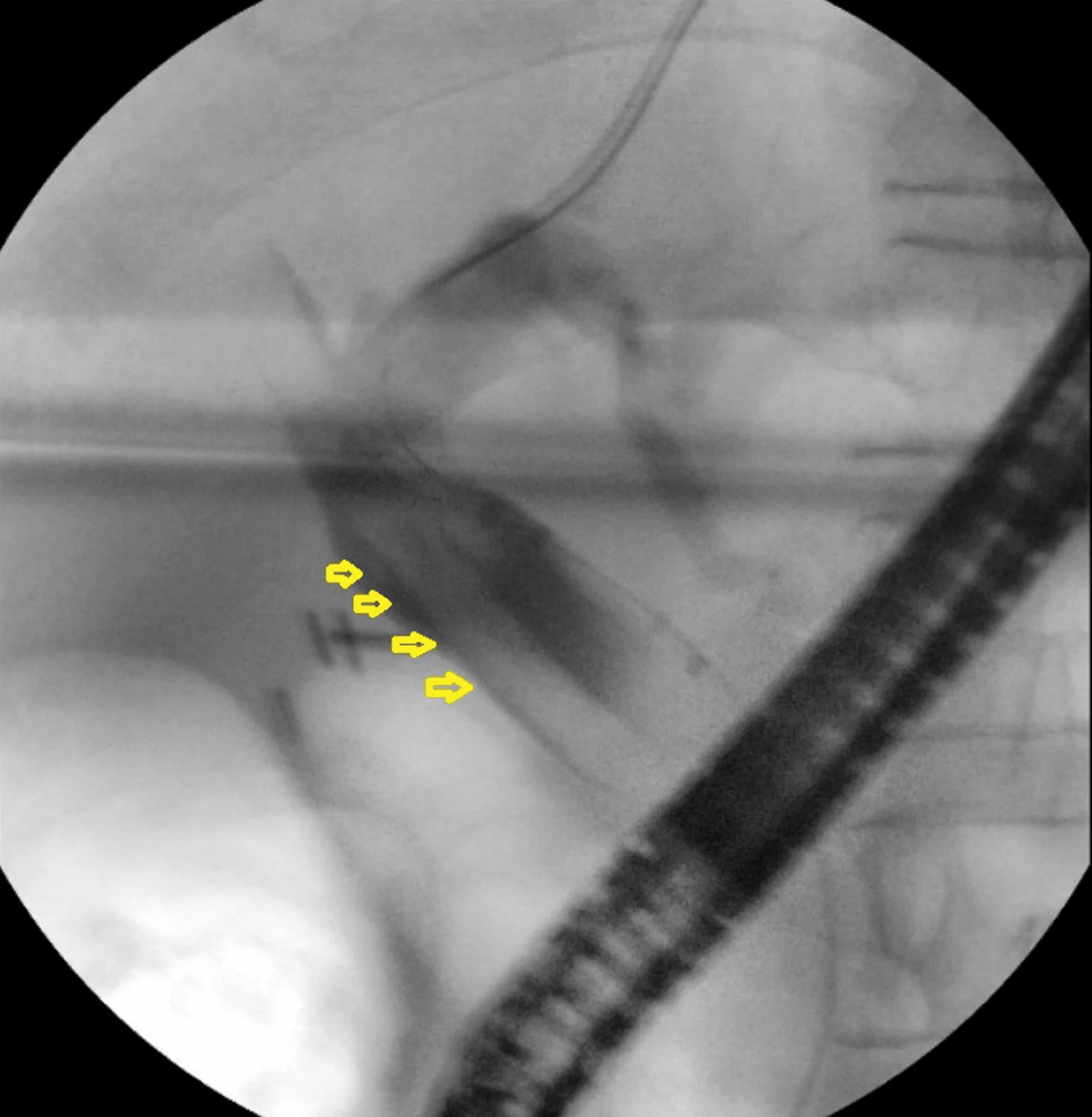
Pressure cholangiogram showing linear filling defect extending within CBD extending from right intrahepatic duct Yellow arrow showing linear filling defect.

**Figure 4:**
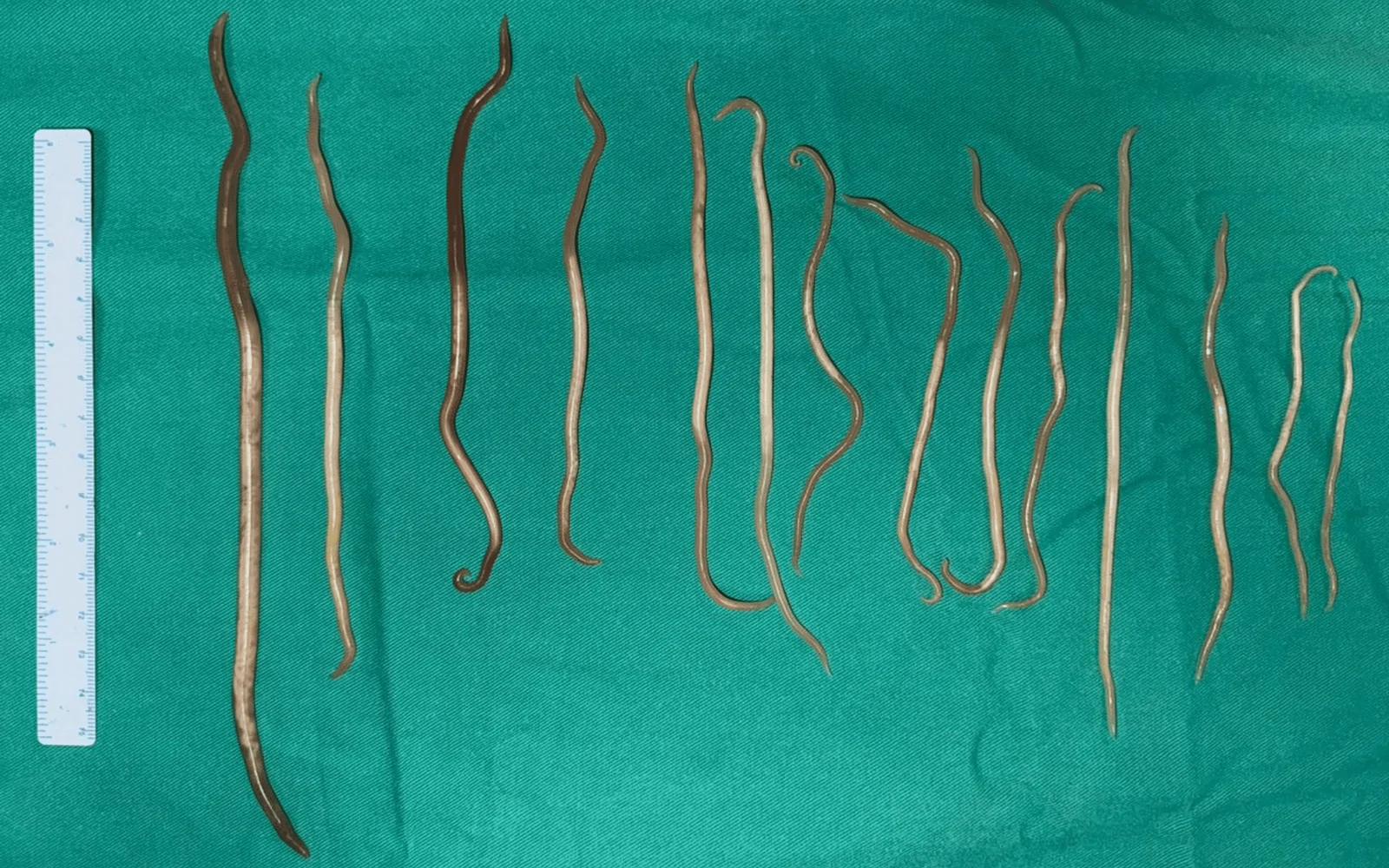
Retrieved worms from the CBD and duodenum CBD: common bile duct

ERCP confirmed the presence of massive numbers of live *A. lumbricoides* in the duodenum and a large one extending within the CBD through the ampulla of Vater (Figure 2). Cannulation was performed with a balloon catheter without additional sphincterotomy, and cholangiography showed a dilated CBD (1.8 cm), with an elongated filling defect extending from the right intrahepatic duct distally (Figure 3). The ampulla was widened with a controlled radial expansion (CRE) balloon to facilitate worm extraction. The worm was successfully extracted using occlusive balloon trawling to the duodenum, and a wire snare was used to hold the worm and extracted with an endoscope. A completion cholangiogram showed no residual filling defects. A total of 14 *A. lumbricoides* were extracted during this procedure (Figure 4). An immediate symptomatic relief was observed post ERCP. 

A multidisciplinary approach involving the Infectious Disease Unit, with treatment strategy of oral albendazole 400 mg OD for three days, and six-monthly dosing was chosen. She was able to be discharged well after two days of admission. Upon follow-up, she remains asymptomatic and was scheduled for six-monthly dosing of albendazole at her local clinic.

## Discussion

*A. lumbricoides* is the largest intestinal nematode infecting humans and has no significant animal reservoir. It is estimated to affect approximately 819 million people worldwide [1], with about 126 million cases reported in Southeast Asia [2]. The infection is endemic in warm, humid equatorial regions, with the highest prevalence observed in China, India, and Southeast Asia [1]. As Malaysia is a tropical country endemic for ascariasis, our population remains at risk, as demonstrated by this case. However, there is no available demographic evidence to link farmers with the degree of infestation.

Infestation is more prevalent among individuals of lower socioeconomic status and those residing in rural areas [1-3]. Our patient demonstrated similar risk factors, as she was involved in agricultural activities with frequent exposure to soil as part of her primary source of income. This might explain the cause of her recurrent presentation.

In its life cycle, migration of adult worms into the CBD may result in biliary obstruction, manifesting as biliary colic, obstructive jaundice, or cholangitis [1]. Our patient similarly presented with biliary colic caused by the presence of nematodes within the CBD due to the worm migration. Previous sphincterotomy may ease the migration to the CBD also. Cholangitis was not a concern due to no associated symptoms of fever or right hypochondriac tenderness.

Abdominal ultrasonography serves as the primary diagnostic modality for biliary ascariasis. Typical findings include a long, slender echogenic tubular structure within the CBD, with or without visible movement [3-5]. In our case, the diagnosis was established using ultrasonography together with the patient's previous history of biliary ascariasis, without the need for advanced imaging. However, ultrasonography may not detect the presence of *A. lumbricoides* in the duodenum [5]. Therefore, a personalized approach to each patient is needed.

In cases where ultrasonography is inconclusive, as distal CBD may be obscured by bowel gas, MRCP has been shown to be superior imaging modality without the risk of contrast allergy and invasiveness. In thin slab imaging, MRCP demonstrates a worm in the CBD as linear hypointense filling defects [6,7]. Nevertheless, MRCP was not pursued in this case due to financial limitations and restricted accessibility resulting from a prolonged waiting list.

CT is another choice for imaging, which shows "bull's eye" appearances in the presence of *A. lumbricoides* infestation [7]. As ultrasonography is available with reliable findings, other advanced imaging modalities were deemed unnecessary in our patient.

Another invasive option is endoscopic ultrasonography, which can demonstrate the nematode in the CBD appearing as a long echogenic structure with a central anechoic linear defect, without any shadow effect [8]. However, due to equipment limitations, this modality was not available in our center.

ERCP plays both a diagnostic and therapeutic role, enabling visualization of filling defects within the biliary tree and facilitating worm extraction using techniques such as balloon trawling or snare retrieval [4,5]. Our patient underwent ERCP without any complications. Despite the availability of ERCP, certain centers may not have the expertise and equipment to perform one. Conservative treatment with antihelminthics may be considered; however, cases needing emergent ERCP should be transferred to a center with ERCP modality [3,9]. A patient with persistent symptoms, obstructive jaundice, or developing cholangitis should be considered for urgent ERCP. Our patient experienced immediate symptomatic relief post ERCP.

Even in cases of heavy *A. lumbricoides* infestation, ERCP remains the treatment of choice in biliary ascariasis [4,5,9]. This can be observed from our patient, as this was not her first episode requiring endoscopic worm extraction.

In preventing recurrent biliary ascariasis, a longer follow-up in patients with a background of low socioeconomic status has proven to be beneficial [3]. Patra et al. reported this as an independent risk factor for recurrent biliary events. Our patient demonstrates recurrent illness with a single dosage of oral albendazole 400 mg OD before, as to why we planned for a daily dosage for three days and a six-monthly oral albendazole 400 mg OD as a means for prophylaxis due to the patient's primary income involved agricultural activities and background of low socioeconomic status. Other factors, such as improving sanitation and educating personal hygiene, will help in preventing infestation [9].

## Conclusions

Biliary ascariasis should be considered in patients from endemic rural or agricultural settings presenting with right upper quadrant pain, even in those with a history of cholecystectomy. This case demonstrates that recurrent biliary ascariasis may occur despite previous ERCP and successful worm extraction, likely reflecting continued environmental exposure and, potentially, altered biliary anatomy following endoscopic sphincterotomy.

Despite the extraction of 14 *A. lumbricoides *worms, standard management principles remained unchanged. ERCP provided definitive diagnosis, complete biliary clearance, and immediate symptomatic relief, demonstrating its effectiveness even in the presence of a heavy biliary worm burden. Patients with recurrent biliary ascariasis should receive appropriate adjunctive antihelminthic therapy together with individualized follow-up, such as repeat ultrasonography and reinfection-prevention measures, for example, improving sanitation and personal hygiene, particularly in endemic communities where ongoing environmental exposure increases the risk of recurrence.
